# CD44 Gene Polymorphisms on Hepatocellular Carcinoma Susceptibility and Clinicopathologic Features

**DOI:** 10.1155/2014/231474

**Published:** 2014-05-27

**Authors:** Ying-Erh Chou, Ming-Ju Hsieh, Hui-Ling Chiou, Hsiang-Lin Lee, Shun-Fa Yang, Tzy-Yen Chen

**Affiliations:** ^1^Institute of Medicine, Chung Shan Medical University, 110 Chien-Kuo North Road, Section 1, Taichung 402, Taiwan; ^2^Cancer Research Center, Changhua Christian Hospital, Changhua 500, Taiwan; ^3^School of Medical Laboratory and Biotechnology, Chung Shan Medical University, Taichung 402, Taiwan; ^4^Department of Surgery, Chung Shan Medical University Hospital, Taichung 402, Taiwan; ^5^Department of Medical Research, Chung Shan Medical University Hospital, Taichung 402, Taiwan; ^6^School of Medicine, Chung Shan Medical University, Taichung 402, Taiwan; ^7^Department of Internal Medicine, Chung Shan Medical University Hospital, Taichung 402, Taiwan

## Abstract

Hepatocellular carcinoma (HCC) is the second leading cause of cancer deaths in Taiwan. CD44, one of the well-known tumor markers, plays an essential role in tumor cell differentiation, invasion, and metastasis. We investigated the CD44 single-nucleotide polymorphisms (SNPs) with environmental risk factors related to HCC susceptibility and clinicopathological characteristics. Six SNPs of CD44 were analyzed using a real-time polymerase chain reaction (PCR) in 203 patients with HCC and in 561 cancer-free controls. We determined that the individuals carrying at least one G allele at CD44 rs187115 has higher risk of developing HCC than did wild-type (AA) carriers. We further observed that the CD44 rs187115 polymorphisms with at least one G allele had a higher frequency of distribution in nonsmoking stage III/IV HCC patients, compared with wild-type carriers. Our results suggested that patients with CD44 rs187115 variant genotypes (AG+GG) were associated with a higher risk of HCC development and that these patients might possess chemoresistance, causing more likely progression to late-stage HCC than wild-type carriers without the overexpression of CD44 induced by heavy smoking. CD44 rs187115 might be involved in CD44 isoform expression of p53 stress response in HCC and provide a marker for predicting worst-case prognosis of HCC.

## 1. Introduction

Hepatocellular carcinoma (HCC), the fifth most common malignancy and the third most lethal type of cancer worldwide, is the second leading cause of cancer-related deaths in Taiwan [1, 2]. The carcinogenesis of HCC is a multistep and complex process. Multiple risk factors, including chronic hepatitis B virus (HBV) or hepatitis C virus (HCV) infection, carcinogen exposure, cirrhosis, and a variety of single-nucleotide polymorphisms (SNPs), are considered to contribute to hepatocarcinogenesis [2–5]. The treatment options for early-stage HCC are surgical resection and liver transplantation. However, because of frequent intrahepatic spread, high level of tumor invasiveness, extrahepatic metastasis, and chemotherapy resistance, the prognosis of HCC remains poor and stable [6].

CD44 is a major adhesion molecule of the extracellular matrix. CD44 glycoproteins are members of the hyaluronate receptor that are associated with many fundamental biological and physiological processes, including embryonal development, lymphocyte homing, inflammation, hematopoiesis, wound healing, apoptosis, and cell migration [7–9]. Although CD44 proteins are involved in the regulation of various cellular processes, CD44 has been indicated to play a pivotal role in tumor cell differentiation, invasion, and metastasis [10, 11]. CD44 has also been identified as one of the well-known markers of breast-cancer-initiating cells (BCICs) [11, 12]. Positive expression of CD44, either individually or combined with other markers, has been observed in cells involved in tumor progression and metastasis, and these cells have been suggested to be cancer stem cells (CSCs) [9, 13–18]. CD44^+^ cells in HCC have been suggested to be involved in the epithelial-mesenchymal transition (EMT), which is a genetic process associated with cancer invasion and metastasis [19–24]. In addition, CD44^+^ cells engraft at high frequencies in mice and appear to possess enhanced chemoresistance [12, 14, 25]. Although the regulation of CD44 expression in hepatocellular carcinoma is not completely understood, recent studies have revealed that the increased CD44 expression in HCC is correlated with increased metastasis, recurrence, resistance to chemotherapy or radiation therapy, and decreased survival [26–28].

Single-nucleotide polymorphisms (SNPs) are the most common type of DNA sequence variation. It occurs when a single nucleotide in the shared sequence of a gene differs between members of a species or in chromosomes. Expression of a gene can be affected by an SNP located within the promoter or other regulatory regions of the gene, which is associated with the occurrence and development of a certain disease [29–32]. Recent studies have suggested the pivotal role of CD44 in HCC [26, 27], and the effect of CD44 polymorphisms on human cancer susceptibility has been documented and described in various cancer studies [33–37]. However, the information for the CD44 SNP expression in HCC is not thoroughly established. Therefore, to elucidate the complex process of hepatocarcinogenesis and improve the scientific basis for preventive interventions, the identification of an SNP or combined interaction of several SNPs in certain genes related to HCC might be helpful, and we hypothesized that CD44 polymorphisms play an essential role in HCC development.

CD44 in human cancer metastasis or prognosis has been well documented, but CD44 gene SNPs and the environmental carcinogens in HCC susceptibility and clinical features remain poorly investigated. In this study, we conducted a case-control study of 6 SNPs, located in the 3′UTR or promoter region of CD44, to analyze the contribution of the 6 polymorphisms of CD44 and the associations of environmental factors and susceptibility or pathological development to/with HCC.

## 2. Material and Methods

### 2.1. Subjects Selection

This study included 561 healthy controls and 203 hepatocellular carcinoma patients. The 561 ethnic group-matched individuals were enrolled as the controls that entered the physical examination at the same hospital. These control groups had no self-reported history of cancer of any site. Personal information and characteristics collected from the study subjects using interviewer-administered questionnaires contained questions involving demographic characteristics and the status of cigarette smoking and alcohol drinking. We collected these hepatocellular carcinoma patients' age, gender, clinical stage, and pathologic TNM stage and tumor differentiation as clinicopathologic characteristics for further analysis. And we also collected the laboratory status such as *α*-fetoprotein, AST, ALT, and AST/ALT ratio for further analysis. Before commencing the study, approval was obtained from the Institutional Review Board of Chung Shan Medical University Hospital, and informed written consent was obtained from each individual.

### 2.2. Selection of CD44 Polymorphisms

A total of six SNPs in CD44 were selected from the International HapMap Project data for this study. We included the SNP rs1425802 in the promoter region. Three SNPs (rs11821102, rs10836347, and rs13347) which locate in the 3′UTR of CD44 were selected in this study since these SNPs were found to affect binding ability of certain microRNA in a Chinese population [37]. Furthermore, the other SNPs (rs187115 and rs713330) were selected in this study because the gene polymorphisms of these SNPs have been found to associate with gastric and breast cancers [36, 37].

### 2.3. DNA Extraction

We collected the whole blood samples from healthy controls and hepatocellular carcinoma patients with tubes containing EDTA; then, the blood samples were centrifuged and stored at −20°C. The venous blood from each subject was drawn into vacutainer tubes containing EDTA and stored at 4°C. Genomic DNA was extracted by QIAamp DNA blood mini kits (Qiagen, Valencia, USA) according to the manufacturer's instructions, and the DNA was dissolved in TE buffer (10 mM Tris (PH 7.8), 1 mM EDTA) and then quantitated by measurement of OD260 [38]. Final DNA preparation was stored at −20°C and used as templates for the following experiments.

### 2.4. Real-Time PCR

Allelic discrimination of the rs1425802, rs187115, rs713330, rs11821102, rs10836347, and rs13347 polymorphisms of the CD44 gene was assessed with the ABI StepOne Real-Time PCR System (Applied Biosystems, Foster City, CA, USA) and analyzed with SDS version 3.0 software (Applied Biosystems) using the TaqMan assay. The final volume for each reaction was 5 *μ*L, containing 2.5 *μ*L TaqMan Genotyping Master Mix, 0.125 *μ*L TaqMan probe mix, and 10 ng genomic DNA. The real-time PCR included an initial denaturation step at 95°C for 10 min, followed by 40 cycles at 95°C for 15 s and then at 60°C for 1 min [39]. For each assay, appropriate controls (nontemplate and known genotype) were included in each typing run to monitor reagent contamination and as a quality control. To validate results from real-time PCR, around 5% of assays were repeated, and several cases of each genotype were confirmed by the DNA sequence analysis.

### 2.5. Statistical Analysis

The distributions of demographic characteristics and genotype frequencies between cases and controls in different genotypes were analyzed by Chi-square test, and Fisher's exact test were using at small sample size was present in some categories of variables. Student's *t*-test was used to estimate laboratory status between the two groups. The odds ratios (ORs) and their 95% confidence intervals (CIs) of the association between genotype frequencies and hepatocellular carcinoma were estimated by multiple logistic regression models, also controlling for covariates. The *P* value of less than 0.05 was considered significant. The data were analyzed on SPSS 12.0 statistical software.

## 3. Results

We analyzed the demographic characteristics of sample specimens and observed that 38.1% (214 of 561) and 34.0% (69 of 203) of healthy controls and patients with HCC, respectively, had consumed alcohol. In addition, 39.2% (220 of 561) and 39.4% (80 of 203) of healthy controls and patients with HCC, respectively, had smoked. The distributional differences of alcohol (*P* = 0.293) and tobacco consumption (*P* = 0.961) between healthy controls and patients with HCC were nonsignificant, whereas age distribution (control: 51.81 ± 14.71; HCC: 64.67 ± 11.81) (*P* < 0.001) and gender distribution (*P* = 0.001) between the 2 subgroups were significantly different (Table 1). To reduce the possible interference of the confounding variables, we used adjusted odds ratios (AORs) with 95% confidence intervals (CIs) that were estimated using multiple logistic regression models after controlling for age and gender in each comparison.

The genotype distributions and associations between HCC and CD44 gene polymorphisms are shown in Table 2. In our recruited control group, the frequencies of CD44, rs187115 (*χ* ^2^ value: 0.29), rs713330 (*χ* ^2^ value: 2.95), rs11821102 (*χ* ^2^ value: 1.15), rs10836347 (*χ* ^2^ value: 2.05), and rs13347 (*χ* ^2^ value: 0.01), were in a Hardy-Weinberg equilibrium, respectively, except for rs1425802 (*χ* ^2^ value: 12.9). The alleles with the highest distribution frequency for rs1425802, rs187115, rs713330, rs11821102, rs10836347, and rs13347, in both healthy controls and recruited HCC patients, respectively, were heterozygous for A/G, homozygous for A/A, homozygous for T/T, homozygous for G/G, homozygous for C/C, and heterozygous for C/C. After adjusting for several variables, HCC individuals with rs1425802, rs713330, rs11821102, rs10836347, and rs13347 polymorphisms of the CD44 gene showed no significant difference compared to wild-type (WT) individuals. However, patients with the CD44 polymorphic rs187115 AG, GG, and AG+GG showed 1.488-fold (95% CI: 1.023–2.291), 4.501-fold (95% CI: 1.687–12.012), and 1.714-fold (95% CI: 1.139–2.579) higher risks of HCC, respectively, compared with wild-type individuals.

To clarify the role of CD44 rs187115 gene polymorphisms in the clinicopathologic status of HCC patients, the distribution frequency of clinical statuses and frequency of CD44 genotypes in HCC patients were estimated, including TNM clinical staging, primary tumor size, lymph node involvement, distant metastasis, hepatitis B surface antigen (HBsAg), antibody to HCV (anti-HCV), and liver cirrhosis. No significant association was observed between the CD44 rs187115 gene polymorphisms and the clinicopathologic status (Table 3). However, when these HCC patients were classified into smoking and nonsmoking groups, a significant association between CD44 rs187115 functional variant “G” and stage III/IV nonsmoking HCC patients was observed (Table 4).

AFP, AST, and ALT are common clinical pathological markers of HCC. In this study, we also analyzed the levels of these pathological markers associated with CD44 genotypic frequencies to clarify the relationship between the progress of the clinical status and the level of clinical pathological markers in HCC patients.  Table 5 shows the associations of CD44 genotypic frequencies with HCC laboratory status, and no significant association was observed between the rs1425802, rs187115, rs713330, rs11821102, rs10836347, and rs13347 gene polymorphisms.

## 4. Discussion

This paper provides novel information on the effects of SNPs of CD44 on HCC susceptibility, interactions with environmental risk factors, and association with clinicopathologic statuses. Cumulative evidence has suggested that progressive genomic changes cause the cellular phenotype to progress from the preneoplastic stage to HCC [40]. Various gene polymorphisms have been identified as being correlated with HCC development [2, 41–43]. Multiple gene alterations, including allelic deletion, insertion, polymorphism mutation, and methylation change, are marked in HCC, causing genetic and molecular aberrations. Thus, genetic components might play an essential role in HCC occurrence. Genetic information in HCC patients compared with healthy controls without HCC is therefore valuable in marking a target gene for the purpose of predicting pathological development and risk of HCC.

Through alternative mRNA splicing, cells produce protein isoforms of CD44, the CD44 standard isoform (CD44s) and the variant form (CD44v). The role of CD44s and CD44v expression in hepatocellular carcinoma remains elusive. Endo and Terada indicated that aberrant expression of CD44s and CD44v (CD44v5, CD44v6, CD44v7-8, and CD44v10) was correlated with poor prognosis in HCC, and a link between CD44v6 and high p53 expression in HCC was also suggested in the study, as determined using immunohistochemical analysis [44]. Yang et al. introduced CD44 as a tumor-initiating cell (TIC) with CD90, and CD44s was observed to be the most frequent TIC marker occurring with other frequent markers including CD24, CD34, CD90, CD133, ALDH, and EpCAM [18]. Recent studies have suggested that the CD44s is highly correlated with the EMT phenotype and with poor prognosis for HCC patients, and CD44s signals the acquisition of a mesenchymal phenotype regulating anchorage-independent capacity in HCC [26, 27]. Previous studies have revealed that the dominant form of CD44 isoforms in various tumors varies according to the location of the cancer cells. The CD44s regulates the mesenchymal phenotype cells, and aberrant CD44v6 expression has been suggested to be correlated with p53 overexpression in HCC [26, 27, 44]. In breast cancer, CD44s was suggested to play a vital role in the response of TGF-*β* during EMT, and the gain of CD44s expression was synchronized with a loss of expression of the variants forms [45]. In lung and colon cancers, high levels of CD44v were proposed as a metastatic tumor marker [46, 47]. Consistent with these results, we observed different CD44 SNP expression in breast cancer and HCC. The variety of dominant CD44 isoforms expressed in different cancers might be responsible for this phenomenon.

Hepatitis virus infection is correlated with elevated oxidative stress in liver cells, leading to DNA changes and instability, increasing the potential risk of developing cirrhosis and/or HCC [48–51]. To interpret the correlations between the HCC clinical status and the CD44 rs187115 genetic variant, we compared the CD44 rs187115 genotypic frequencies with the clinical status in 203 HCC patients. However, no significant association was observed (Table 3). However, we observed a significant association with the CD44 rs187115 polymorphism in 123 nonsmoking stage III/IV HCC patients (Table 4). Heavy smoking or chronic cigarette smoke exposure was associated with CD44 overexpression and EMT occurrence. Regarding oral cancer, previous studies have suggested a statistically significant association between smoking and CD44 expression in SCCs located in the oropharynx, hypopharynx, and larynx [52]. Chronic exposure to cigarette smoke causes the emergence of cell populations bearing markers of self-renewing stem-like cells in breast cancer, including CD44^+^ cells [53]. A recent study indicated that smoking history and quantity are risk factors for HBV-related HCC recurrence and liver-specific mortality (LSM) of patients after surgery [54]. However, the correlations between CD44 expression and tobacco smoking in HCC are still not completely understood. In certain genes, an SNP arising in the coding, promoter, or regulatory region might have functional consequences [29]. The CD44 SNP rs187115 was located in the first intron of CD44. Although no regulatory role of intron1 of CD44 has been proposed, previous studies have suggested a possible role of CD44 rs187115 functional variants with chemoresistance and cellular stress response in a p53-dependent manner [32]. In this study, we observed the CD44 rs187115 AG+GG phenotypes distributed in stage III/IV HCC patients who did not smoke and CD44 rs187115 functional variants involved in chemoresistance and p53 stress response. It is possible that even without the overexpression of CD44 induced by heavy smoking some crucial factors of the p53 signaling pathway are affected by CD44 rs187115 functional variants, which ultimately contributes to CD44 misregulation, resulting in chemoresistance and a poor cancer prognosis. Previous studies have proposed that it is not the mutation of CD44 but those factors promoting carcinogenesis that control the patterns of the misregulated CD44 in most cancers [8]. For example, the alternative splicing of CD44 controlled by the mitogenic signals, including the Ras-MAP cascade [55, 56], and the loss of various subunits of the SWI/SNF chromatin remodeling complex result in the loss of CD44 transcription [57, 58]. Because the aberrant CD44v6 expression is suggested to be associated with high levels of p53 expression in HCC [44], CD44 rs187115 functional variants might shed light on determining the correlations between CD44v6 and p53 overexpression in hepatocellular carcinoma. However, the underlying mechanism of CD44 regulation in HCC, particularly the functions of SNPs in the first intron of CD44 to p53 stress response, requires further well-designed study to clarify its role in tumor aggressiveness and CSCs.

In conclusion, our study first demonstrated a significant association between the CD44 rs187115 A/G polymorphism and risk of HCC. Patients who carry the CD44 rs187115 functional variant G might possess chemoresistance and be more likely to progress to late-stage HCC than those with the wild-type carriers without the overexpression of CD44 induced by tobacco smoking. Our results showed that the CD44 genetic variants play a significant role in p53 stress response and affect tumor incidence and survival. CD44 rs187115 might serve as a marker to predict poor prognosis in HCC patients.

## Figures and Tables

**Table 1:** The distributions of demographical characteristics in 561 controls and 203 patients with HCC.

Variable	Controls (*N* = 561)	Patients (*N* = 203)	*P* value
Age (yrs)			
Mean ± S.D.	51.81 ± 14.71	64.67 ± 11.81	*P* < 0.001*
Gender	*n* (%)	*n* (%)	
Male	457 (81.5%)	141 (69.5%)	
Female	104 (18.5%)	62 (30.5%)	*P* = 0.001*
Alcohol consumption			
No	347 (61.9%)	134 (66.0%)	
Yes	214 (38.1%)	69 (34.0%)	*P* = 0.293
Tobacco consumption			
No	341 (60.8%)	123 (60.6%)	
Yes	220 (39.2%)	80 (39.4%)	*P* = 0.961
Stage			
I + II		127 (62.6%)	
III + IV		76 (37.4%)	
Tumor T status			
≤T2		129 (63.5%)	
>T2		74 (36.5%)	
Lymph node status			
N0		194 (95.6%)	
N1 + N2		9 (4.4%)	
Metastasis			
M0		192 (94.6%)	
M1		11 (5.4%)	

Mann-Whitney *U* test or Fisher's exact test was used between healthy controls and patients with HCC. *P value < 0.05 is statistically significant.

**Table 2:** Distribution frequency of CD44 genotypes in 561 healthy controls and 203 patients with HCC.

Variable	Controls (*N* = 561) *n* (%)	Patients (*N* = 203) *n* (%)	OR (95% CI)	AOR (95% CI)
rs1425802				
AA	194 (34.6%)	70 (34.5%)	1.00	1.00
AG	235 (41.9%)	75 (36.9%)	0.884 (0.607–1.290)	0.763 (0.487–1.194)
GG	132 (23.5%)	58 (28.6%)	1.218 (0.806–1.839)	1.071 (0.659–1.741)
AG+GG	367 (65.4%)	133 (65.5%)	1.004 (0.717–1.408)	0.878 (0.588–1.310)
rs187115				
AA	403 (71.8%)	123 (60.6%)	1.00	1.00
AG	143 (25.5%)	66 (32.5%)	1.512 (1.061–2.156)*	1.488 (1.023–2.291)*
GG	15 (2.7%)	14 (6.9%)	3.058 (1.436–6.512)*	4.501 (1.687–12.012)*
AG+GG	158 (28.2%)	80 (39.4%)	1.659 (1.185–2.322)*	1.714 (1.139–2.579)*
rs713330				
TT	467 (83.2%)	167 (82.3%)	1.00	1.00
TC	86 (15.4%)	36 (17.7%)	1.171 (0.764–1.795)	0.945 (0.564–1.585)
CC	8 (1.4%)	0 (0%)	—	—
TC+CC	94 (16.8%)	36 (17.7%)	1.071 (0.702–1.635)	0.897 (0.537–1.498)
rs11821102				
GG	481 (85.7%)	173 (85.2%)	1.00	1.00
GA	75 (13.4%)	29 (14.3%)	1.075 (0.677–1.707)	1.126 (0.644–1.970)
AA	5 (0.9%)	1 (0.5%)	0.556 (0.065–4.793)	0.630 (0.064–6.165)
GA+AA	80 (14.3%)	30 (14.8%)	1.043 (0.662–1.642)	1.091 (0.631–1.885)
rs10836347				
CC	487 (86.8%)	180 (88.7%)	1.00	1.00
CT	69 (12.3%)	23 (11.3%)	0.902 (0.546–1.490)	0.899 (0.494–1.634)
TT	5 (0.9%)	0 (0%)	—	—
CT+TT	74 (13.2%)	23 (11.3%)	0.841 (0.511–1.384)	0.872 (0.481–1.582)
rs13347				
CC	295 (52.6%)	110 (54.2%)	1.00	1.00
CT	223 (39.8%)	72 (35.5%)	0.866 (0.614–1.222)	0.859 (0.568–1.300)
TT	43 (7.6%)	21 (10.3%)	1.310 (0.744–2.306)	1.357 (0.694–2.653)
CT+TT	266 (47.4%)	93 (45.8%)	0.938 (0.679–1.294)	0.944 (0.642–1.388)

The odds ratios (ORs) with their 95% confidence intervals (CIs) were estimated by logistic regression models. The adjusted odds ratios (AORs) with their 95% confidence intervals (CIs) were estimated by multiple logistic regression models after controlling for age and gender. *P value < 0.05 is statistically significant.

**Table 3:** Adjusted odds ratio (AOR) and 95% confidence interval (CI) of clinical status and CD44 rs187115 genotypic frequencies in 203 HCC patients.

Variable	Genotypic frequencies			
	AA (*N* = 123)	AG+GG (*N* = 80)	OR (95% CI)	AOR (95% CI)
	*n* (%)	*n* (%)		
Clinical stage				
Stage I/II	81 (65.9%)	46 (57.5%)	1.00	1.00
Stage III/IV	42 (34.1%)	34 (42.5%)	1.425 (0.799–2.544)	1.754 (0.818–3.760)
Tumor size				
≦T2	81 (65.9%)	48 (60.0%)	1.00	1.00
>T2	42 (34.1%)	32 (40.0%)	1.286 (0.718–2.301)	1.375 (0.639–2.960)
Lymph node metastasis				
No	120 (97.6%)	74 (92.5%)	1.00	1.00
Yes	3 (2.4%)	6 (7.5%)	3.243 (0.787–13.362)	5.024 (0.892–28.309)
Distant metastasis				
No	118 (95.9%)	74 (92.5%)	1.00	1.00
Yes	5 (4.1%)	6 (7.5%)	1.914 (0.564–6.494)	3.360 (0.600–18.226)
The Child-Pugh grade				
A	89 (72.4%)	58 (72.5%)	1.00	1.00
B or C	34 (27.6%)	22 (27.5%)	0.993 (0.529–1.864)	1.208 (0.534–2.731)
HBsAg				
Negative	73 (59.3%)	46 (57.5%)	1.00	1.00
Positive	50 (40.7%)	34 (42.5%)	1.079 (0.610–1.910)	1.153 (0.538–2.471)
Anti-HCV				
Negative	67 (54.5%)	40 (50.0%)	1.00	1.00
Positive	56 (45.5%)	40 (50.0%)	1.196 (0.681–2.103)	1.075 (0.526–2.198)
Liver cirrhosis				
Negative	30 (24.4%)	26 (32.5%)	1.00	1.00
Positive	93 (75.6%)	54 (67.5%)	0.670 (0.359–1.249)	0.500 (0.220–1.133)

The ORs analyzed by their 95% CIs were estimated by logistic regression models.

The AORs with their 95% CI were estimated by multiple logistic regression models, after controlling for age, gender, and tobacco and alcohol consumption.

>T2: multiple tumor more than 5 cm or tumor involving a major branch of the portal or hepatic vein(s).

**Table 4:** Adjusted odds ratio (AOR) and 95% confidence interval (CI) of clinical status and CD44 rs187115 genotypic frequencies in 123 HCC patients without smoking.

Variable	Genotypic frequencies			
	AA (*N* = 74)	AG+GG (*N* = 49)	OR (95% CI)	AOR (95% CI)
	*n* (%)	*n* (%)		
Clinical stage				
Stage I/II	55 (74.3%)	28 (57.1%)	1.00	1.00
Stage III/IV	19 (25.7%)	21 (42.9%)	2.171 (1.006–4.687)*	2.259 (1.036–4.924)*
Tumor size				
≦T2	55 (74.3%)	31 (63.3%)	1.00	1.00
>T2	19 (25.7%)	18 (36.7%)	1.681 (0.770–3.669)	1.441 (0.409–5.077)
Lymph node metastasis				
No	73 (98.6%)	45 (91.8%)	1.00	1.00
Yes	1 (1.4%)	4 (8.2%)	6.489 (0.703–59.899)	6.876 (0.308–153.563)
Distant metastasis				
No	72 (97.3%)	44 (89.8%)	1.00	1.00
Yes	2 (2.7%)	5 (10.2%)	4.091 (0.761–21.998)	16.533 (0.848–322.266)
The Child-Pugh grade				
A	54 (73.0%)	36 (73.5%)	1.00	1.00
B or C	20 (27.0%)	13 (26.5%)	0.975 (0.431–2.204)	0.918 (0.244–3.455)
HBsAg				
Negative	45 (60.8%)	29 (59.2%)	1.00	1.00
Positive	29 (39.2%)	20 (40.8%)	1.070 (0.512–2.235)	1.229 (0.293–5.160)
Anti-HCV				
Negative	42 (56.8%)	25 (51.0%)	1.00	1.00
Positive	32 (43.2%)	24 (49.0%)	1.260 (0.610–2.601)	1.228 (0.342–4.410)
Liver cirrhosis				
Negative	15 (20.3%)	17 (34.7%)	1.00	1.00
Positive	59 (79.7%)	32 (65.3%)	0.479 (0.211–1.083)	0.295 (0.077–1.133)

The ORs analyzed by their 95% CIs were estimated by logistic regression models.

The AORs with their 95% CI were estimated by multiple logistic regression models, after controlling for age, gender, and alcohol consumption.

>T2: multiple tumor more than 5 cm or tumor involving a major branch of the portal or hepatic vein(s).

*P value < 0.05 is statistically significant.

**Table 5:** Association of CD44 genotypic frequencies with HCC laboratory status.

Characteristic	*α*-Fetoprotein^a^ (ng/mL)	AST^a^ (IU/L)	ALT^a^ (IU/L)	AST/ALT ratio^a^
rs1425802				
AA	5097.9 ± 2375.8	251.1 ± 61.4	189.7 ± 40.6	1.67 ± 0.22
AG/GG	3799.7 ± 1302.1	154.0 ± 21.6	139.8 ± 21.9	1.46 ± 0.08
*P* value	0.602	0.071	0.238	0.268
rs187115				
AA	2762.4 ± 1326.1	209.4 ± 39.0	183.2 ± 31.4	1.38 ± 0.08
AG/GG	6530.4 ± 2179.8	153.8 ± 24.7	116.8 ± 15.2	1.76 ± 0.19
*P* value	0.119	0.289	0.106	0.077
rs713330				
TT	4326.7 ± 1313.1	183.0 ± 29.0	155.3 ± 23.4	1.59 ± 0.11
TC/CC	3879.2 ± 2717.1	208.4 ± 52.8	165.0 ± 31.9	1.26 ± 0.10
*P* value	0.885	0.705	0.853	0.169
rs11821102				
GG	4866.9 ± 1377.5	200.4 ± 29.5	165.6 ± 22.8	1.56 ± 0.11
GA/AA	674.4 ± 469.4	113.0 ± 29.0	107.3 ± 32.3	1.40 ± 0.12
*P* value	0.208	0.226	0.303	0.550
rs10836347				
CC	4265.0 ± 1241.4	181.7 ± 24.0	144.8 ± 18.1	1.56 ± 0.10
CT/TT	4108.6 ± 3837.3	232.5 ± 127.4	252.6 ± 106.2	1.27 ± 0.23
*P* value	0.967	0.930	0.088	0.317
rs13347				
CC	3964.4 ± 1654.2	157.5 ± 36.9	124.1 ± 24.7	1.64 ± 0.16
CT/TT	4582.0 ± 1684.4	223.0 ± 34.7	195.9 ± 32.3	1.40 ± 0.08
*P* value	0.795	0.203	0.074	0.201

Mann-Whitney *U* test was used between two groups.

^a^Mean ± S.E.
